# Unraveling the Mechanistic Links Between Species Diversity and Infection Risk From Zoonotic Pathogens With Direct Transmission Among Reservoir Hosts: Rodent‐Orthohantavirus Systems as Models

**DOI:** 10.1002/ece3.71597

**Published:** 2025-06-14

**Authors:** Andreas Eleftheriou, Angela D. Luis

**Affiliations:** ^1^ Wildlife Biology Program University of Montana Missoula Montana USA

**Keywords:** community composition, density‐dependent transmission, emerging infectious diseases, population regulation, rodent‐borne zoonoses, species diversity

## Abstract

To explain patterns between anthropogenic loss of species diversity and the rise in the number of novel zoonotic diseases, the “dilution effect” hypothesis predicts that with lower species diversity, infection risk will increase. The underlying mechanisms have been largely investigated in systems where pathogen transmission is vector‐borne or environmental. Relatively less research has been conducted in systems where transmission is direct, such as with orthohantaviruses (hereafter hantaviruses) and their rodent reservoir hosts. These systems are commonly cited as supporting a negative diversity‐disease pattern. To motivate empirical research on underlying mechanisms driving this pattern, we extend a mechanistic framework that links species diversity and infection prevalence of directly transmitted zoonotic pathogens by using rodent‐hantavirus systems in the Americas as models. Additionally, we summarize empirical studies, synthesize mechanistic evidence, and identify knowledge gaps. Our findings suggest that host regulation is a key mechanism likely to drive diversity‐disease patterns in rodent‐hantavirus systems of the Americas. Other mechanisms have received less empirical support but also less attention. Although host regulation likely functions via density‐dependent transmission, and can thus change contact rates among hosts, consequences to other mechanisms have been neglected. As observed in rodent‐hantavirus systems in the Americas, we propose that for a negative diversity‐disease pattern to manifest, the primary reservoir host species should be resilient to anthropogenic disturbance but also vulnerable to competition, predation, or both, and the “diversity” measure should be associated with host density.

## Graphical abstract

In this review, we synthesize mechanistic evidence for diversity‐disease patterns in rodent‐orthohantavirus systems in the Americas, models of directly transmitted disease systems. We found that host regulation has been examined the most while other mechanisms have received less attention. Based on our findings, we propose that for a negative diversity‐disease pattern to manifest, the primary reservoir host should be resilient to anthropogenic disturbance and vulnerable to competition, predation, or both, and the “diversity” measure should be associated with host density.

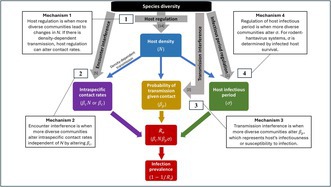

## 1 Human‐Driven Relationship Between Diversity and Disease

Anthropogenic disturbance is driving widespread losses in species diversity (Van Der Wal et al. 2008; Ceballos et al. 2015; Dantas and Fonseca 2024). Parallel to these losses, the number of novel zoonotic diseases is on the rise (Smith et al. 2014; Cunningham et al. 2017). The “dilution effect” hypothesis postulates that as species diversity decreases, infection risk increases (i.e., higher infection prevalence or transmission) through an array of mechanisms that may or may not be dependent on host density (reviewed in Keesing et al. 2006). This hypothesis has attracted substantial attention as it suggests a win‐win scenario for biological conservation and public health. However, its generality and applicability have been debated (Randolph and Dobson 2012; Ostfeld 2013).

Causal mechanisms were instrumental to the original “dilution effect” hypothesis, centered on *Borrelia burgdorferi* (the causative agent of the vector‐borne Lyme disease) that can infect multiple hosts with a range of reservoir competencies, with the white‐footed mouse ( *Peromyscus leucopus* ) being the most competent host (Ostfeld and Keesing 2000). The concept was later expanded to include pathogens with environmental and direct transmission (Keesing et al. 2006). However, most mechanistic studies have focused on pathogens with vector‐borne or environmental transmission (LoGiudice et al. 2003; Orlofske et al. 2012). Fewer mechanistic studies have focused on pathogens with direct transmission, such as orthohantaviruses and mammarenaviruses (Mills 2006; Kilpatrick et al. 2017; Luis et al. 2018).

In recent years, there has been a call to move beyond simple correlations between diversity and disease measures towards investigations of the underlying mechanisms (Johnson et al. 2013; Luis et al. 2018) initially proposed by Keesing et al. (2006). Briefly, the mechanisms by which diversity could affect prevalence of a directly transmitted pathogen with one reservoir host, include encounter reduction, transmission reduction, susceptible host regulation, recovery augmentation, and infected host mortality (Keesing et al. 2006), which we discuss in detail below. Unraveling mechanisms at play is not merely an academic exercise. Firstly, we can discern why a diversity‐disease pattern occurs, which helps avoid emphasizing spurious associations, and secondly, we can incorporate empirically derived mechanisms in predictive models, allowing us to generate more transparent and robust recommendations for targeted interventions.

**FIGURE 1:**
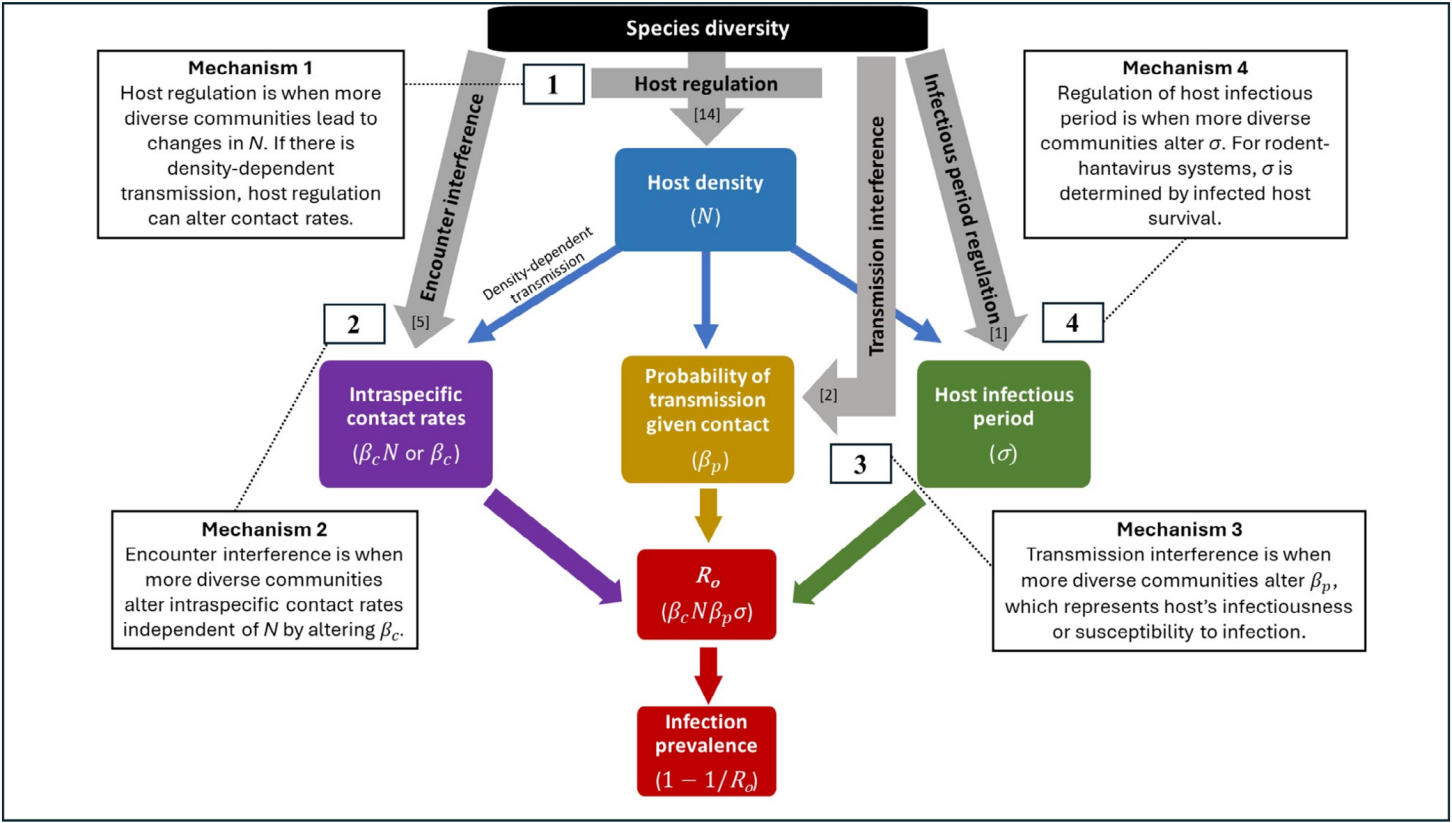
Conceptual diagram describing potential relationships between species diversity and infection prevalence of a directly transmitted pathogen with one primary reservoir host that is persistently infected. Gray arrows propose mechanisms that diversity can act through to affect *R*_0_ components in colored squares (contact rates [$\beta_{c}N$ or *β*_c_], probability of transmission given contact [*β*_p_], and host infectious period [*σ*]) that determine infection prevalence at equilibrium. Diversity can determine prevalence via host regulation (blue arrows), or directly (gray arrows) independent of host regulation (mechanism 1), via encounter interference (mechanism 2), transmission interference (mechanism 3), and infectious period regulation (mechanism 4). When there is density‐dependent transmission, contact rates = $\beta_{c}N$, and *R*_0_ = $\beta_{c}N\beta_{p}$ *σ*. Numbers in brackets illustrate number of studies across rodent‐hantavirus systems with support for each mechanism (studies included in multiple counts if more than one mechanism was examined).

### 1.1 Diversity‐Disease Patterns in Directly Transmitted Disease Systems

Although numerous directly transmitted disease systems exist, we know relatively less about their diversity–disease patterns compared to other disease systems. To date, the research focus has been on orthohantaviruses (*Orthohantavirus*; Hantaviridae; Bunyavirales; hereafter hantaviruses) and their rodent reservoir hosts (Adams et al. 2017) as models. Some attention has also been given to *Mycobacterium* spp. (e.g., Barasona et al. 2019) and mammarenaviruses (Mills 2006).

In directly transmitted disease systems of animals, evidence shows that a negative diversity‐disease pattern occurs more often (Huang et al. 2017), with rodent‐hantavirus systems cited as prime examples (Kilpatrick et al. 2017; Rohr et al. 2020). To understand why we see this principal pattern, it is helpful to switch our focus to the study of mechanisms. Although recent reviews have synthesized evidence about diversity‐disease patterns in rodent‐hantavirus systems (Dearing and Dizney 2010; Jonsson et al. 2010; Heyman et al. 2012; Khalil et al. 2014; Forbes et al. 2018; Guterres and de Lemos 2018), there has been no systematic review delving into the underlying mechanisms, which is our central focus herein.

To present the evidence, we first introduce the mechanisms that can drive diversity‐disease patterns in directly transmitted disease systems by extending the previous framework initially proposed in Keesing et al. (2006). Next, we summarize and synthesize evidence for each mechanism and explain how our conclusions may be generalized and integrated into existing theory. Lastly, we provide recommendations to guide prospective empirical studies.

## 2 Global Rodent‐Hantavirus Systems

Hantaviruses are found in Europe, Asia, Africa, and the Americas (Laenen et al. 2019). Several are emerging zoonoses, including Sin Nombre virus (SNV) and Andes virus (ANDV) in the Americas, Puumala virus (PUUV) in Europe, and Hantaan virus (HTNV) in Asia (Kruger et al. 2015). SNV and ANDV cause the more severe hantavirus cardiopulmonary syndrome whereas HTNV and PUUV cause hemorrhagic fever with renal syndrome (Jonsson et al. 2010; Brocato and Hooper 2019). Humans typically become exposed via inhalation of aerosolized excreta and secreta of infected hosts through indirect transmission (Jonsson et al. 2010).

Unlike in the vector‐borne Lyme disease system where host species vary in reservoir competence (LoGiudice et al. 2003), hantaviruses are typically thought to have one primary reservoir host (Dearing and Dizney 2010). However, the one host‐one hantavirus paradigm has been contested for various systems in the Americas, Europe, and Asia (Milholland et al. 2018). For example, the North American deermouse ( *Peromyscus maniculatus* ) was identified as the primary reservoir of SNV (Childs et al. 1994), but SNV RNA has also been detected from pinyon mice ( *P. boylii* ) and house mice ( *Mus musculus* ) (Goodfellow et al. 2021). Presently, the transmission role of secondary versus primary hosts is unclear and further investigations are necessary to define its significance. Herein, we assume the one host‐one hantavirus paradigm, where one primary host plays the most significant role in hantavirus transmission.

Within host populations, hantaviruses are primarily transmitted during intraspecific aggressive encounters (Jonsson et al. 2010; Warner et al. 2019), although environmental transmission may also occur, mainly in the bank vole‐PUUV disease system in Europe (Kallio et al. 2006). Infection does not lead to clinical disease in hosts (Ermonval et al. 2016), but free‐ranging hosts may have lower survival (Luis et al. 2012) and fecundity (Kallio et al. 2015).

## 3 Diversity‐Disease Mechanisms: Theory

Rodent‐hantavirus studies have mainly used two metrics to quantify infection risk, including prevalence of hantavirus antibody and number of infected individuals. Because most studies used antibody prevalence, we focused on this metric. Antibody prevalence is a justified marker of infection status because hantaviruses cause a chronic infection in their hosts despite life‐long production of antibodies (Jonsson et al. 2010).

### 3.1 Mechanistic Determinants of Infection Prevalence

For directly transmitted diseases with one primary reservoir, such as hantaviruses, there are three determinants of *R*_0_, the basic reproductive number, which describes the mean number of secondary infections from one primary case in an entirely susceptible population (Delamater et al. 2019). *R*_0_ dictates infection prevalence at equilibrium—contact rate, probability of transmission given contact (*β*_p_), and infectious period (*σ*). The contact rate is *β*_c_[*N*] if there is density‐dependent transmission (where *N* denotes host density), and *β*_c_ if there is frequency‐dependent transmission. Often, the product of *β*_c_ and *β*_p_ is collapsed into a single parameter, called the transmission rate, *β*.

If species diversity affects any of these three determinants of *R*_0_, it can drive infection prevalence and lead to a negative or positive diversity‐disease pattern. Therefore, there are four mechanisms that can drive relationships between species diversity and infection prevalence (Figure 1, gray arrows). For density‐dependent transmission, $R_{0}=\beta_{c}N\beta_{p}\sigma$, and for frequency‐dependent transmission $R_{0}=\beta_{c}\beta_{p}\sigma$. For chronic infections, such as hantavirus infections in reservoir hosts, infection prevalence at equilibrium will be $1-\frac{1}{R_{0}}.$

We propose an extension of the mechanistic framework originally introduced in Keesing et al. (2006) for directly transmitted pathogens. Firstly, we revise the definitions of mechanisms so they can be broadly applied to any diversity‐disease pattern. Secondly, we assume chronic infection in the primary reservoir host. Lastly, we expand the pivotal role host density may play in mechanistic interactions.

Mechanism 1 is host regulation, when more diverse communities lead to changes in host density, *N*, for example through competition or mutualism. If there is density‐dependent transmission, host regulation can alter intraspecific contact rates (Figure 1, leftmost blue arrow). Mechanism 2 is encounter interference, when more diverse communities alter intraspecific contact rates independent of *N* by altering *β*_c_, for example through changing behavior that affects home range size. Mechanism 3 is transmission interference, when more diverse communities alter the probability of transmission given contact, *β*_p_, which represents the host's infectiousness or susceptibility to infection, for example by causing stress‐induced immunosuppression and making hosts more susceptible to infection (Dhabhar and Mcewen 1997) or more infectious (Romeo et al. 2020). Mechanism 4 is regulation of host infectious period (infected host mortality, recovery augmentation, or both), when more diverse communities alter *σ*, for example by increasing mortality of infected hosts through competition or predation. For rodent‐hantavirus systems, the infectious period (*σ*) is determined by infected host survival because hosts never recover from infection.

Our extension of the original framework in Keesing et al. (2006) explicitly incorporates how host density and regulation (mechanism 1) can potentially have additional indirect effects on infection prevalence beyond intraspecific contact rates (Figure 1, blue arrows). Evidence suggests that a higher host density could increase transmission probability (*β*_p_), via stress‐induced immunosuppression (e.g., Harper and Austad 2004). Alternatively, a higher host density could reduce transmission probability, if it leads to enhanced immunity (e.g., Wilson et al. 2002). Furthermore, a higher host density could increase the infectious period (*σ*) by extending infected host survival if access to resources increased (e.g., Clutton‐Brock et al. 1999). However, a higher host density could also shorten the infectious period if it leads to a decline in resources and hence, survival (e.g., Mduma et al. 1999). Therefore, whenever we examine relationships between diversity and disease, and diversity can regulate the host population, we must consider whether host regulation could also indirectly affect prevalence via transmission interference, infectious period regulation, or both, in addition to changes in intraspecific contact rates via density‐dependent transmission. It is pivotal to recall that multiple mechanisms, acting alone (Figure 1, gray lines) or via host regulation (Figure 1, blue lines), may be at play at any given time, which may lead to opposing, additive, or synergistic effects on infection outcomes.

## 4 Diversity‐Disease Mechanisms: Evidence

We reviewed the literature through a systematic search using the Google Scholar search engine with the terms: “dilution effect”, “hantavirus”, “disease”, “infection”, “rodent” AND “species diversity” through April 15, 2024 (Figure S1). We identified 21 empirical studies in which diversity‐disease patterns were investigated (Tables S1 and S2). Underlying mechanisms were examined in 15 studies (Table 1). Most attention has been given to host regulation, and to a lesser extent, encounter interference, whereas transmission interference and infectious period regulation have been neglected, reflecting a general trend across systems (Keesing and Ostfeld 2021a). This is likely because host regulation may be easier to study compared to others that may require more complicated methods.

**TABLE 1:** Mechanistic diversity‐disease studies with rodent‐hantavirus systems in the Americas. We list data on the system, diversity‐disease outcome, support (with directionality) for relationships between diversity and host density (*N*) (mechanism = host regulation), diversity and intraspecific contact rates ($\beta_{c}$) (mechanism = encounter interference), diversity and transmission probability ($\beta_{p}$) (mechanism = transmission interference), and diversity and host infectious period (*σ*) (mechanism = infectious period regulation), metrics of diversity and disease, and study reference. The complete table that includes non‐mechanistic studies is found in the Supporting Information.

Host–pathogen system	Disease outcome	Host density (*N*)	Contact rates ($\beta_{c}$)	Transmission probability ($\beta_{p}$)	Host infectious period (σ)	Diversity metric	Disease metric	Study reference
*P. maniculatus* –SNV	D^a^	−			−?	RDY	AP	Clay et al. (2009a)^a^
	N^a^	N	−?			RDY	AP	Clay et al. (2009b)^†^
	N	N				SDY	AP	Dizney and Ruedas (2009)^†^
	D	−				SDY	AP/NI	Lehmer et al. (2012)
	D	N	−			RDY	AP	Dizney and Dearing (2016)
		N	N			RDY		Rubio et al. (2017)
	D^b^	−				SDY	AP/NI	Luis et al. (2018)
	N^c^	N				SDY	AP/NI	Luis et al. (2018)
	CA^b^	NA	+?	+?		SDY	AP	Luis et al. (2018)
	CA^c^	NA	+?	+?		SDY	AP	Luis et al. (2018)
	N^b^	N				RDY	AP	Calisher et al. (2002)^†^
	N^c^	+				NHD	AP	Carver et al. (2011)
			+/NM	+		NHD		Eleftheriou et al. (2021)
*O. longicaudatus* —ANDV	N	N				SDY	AP/NI	Rubio et al. (2019)
*C. laucha* —LGNV	N	N				SDY	AP	Yahnke et al. (2001)^†^
*A. montensis—*JABV	N	N				RDY	AAP	Eastwood et al. (2018)^†^
Various	D	−				RDY	AP	Suzán et al. (2009)
		−				SDY		Suzán et al. (2008)

Abbreviations: Host–Pathogen System: ANDV = Andes virus, BAYV = Bayou virus, JABV = Jabora virus, LGNV = Laguna Negra virus, SNV = Sin Nombre virus. Disease Outcome: A = amplification, CA = component amplification (i.e., not the main effect), D = dilution. Diversity Metric: NHD = nonhost density, RDY = rodent diversity, SDY = small mammal diversity. Disease Metric: AAP = antibody and/or antigen prevalence; AP = antibody prevalence; NI = number of infected hosts. ALL: “.” = not explicitly examined, “+” = positive relationship, “−” = negative relationship, *N* = no relationship, NA = not applicable, NM = non‐monotonic. Studies that share letter superscripts (a–c) used subsets of the same datasets. Question marks denote published relationships that the authors find unclear.

^†^ Includes results from statistical analyses that were not performed by the original study authors. More details in Supporting Information.

Below, we summarize and synthesize the mechanistic evidence to provide a robust foundation for empirical studies. We focus on the deermouse‐SNV system as it was the most investigated and because all three diversity‐disease patterns have been found in this system, which allows us to examine which mechanism(s) may be driving these patterns.

### 4.1 Mechanism 1: Host Regulation

When species diversity regulates the host population, all determinants of *R*_0_—contact rates (*β*_c_[*N*]), transmission probability (*β*_p_), and infectious period (*σ*)—could be affected (Figure 1, blue arrows). Species diversity may regulate the density of the host population, *N*, through changes in birth or mortality rates, via processes such as competition, predation, and mutualism.

There is compelling evidence that host regulation is the key mechanism driving diversity‐disease patterns. As most studies used diversity indices of small mammals, species diversity likely serves as a proxy for heterospecific competitors that may regulate the host population. All studies that identified a negative relationship between diversity and density (“−” in the host density column of Table 1, indicating host regulation) also found a negative diversity‐disease pattern (“D” in the outcome column, Table 1). However, an increase in diversity does not always lead to a reduction in host density (“N” in the host density column of Table 1 indicating no host regulation), and subsequently, no diversity‐disease pattern is generally observed (“N” in the outcome column, Table 1). This likely occurs when the measure of “diversity” is not a proxy for species that regulate the host. For example, Luis et al. (2018) found a negative diversity‐disease pattern in the southwest USA, where they also observed host regulation but found no such evidence in Montana, USA, where host regulation was absent. Thus, diversity‐mediated changes in host density can impact infection outcomes.

However, two studies do not support this conclusion. The first, by Dizney and Dearing (2016) from Utah USA, reported a negative diversity‐disease pattern at two sites varying in diversity but not deermouse density. The second, by Carver et al. (2011) from one site in Montana, USA, reported a “temporal dilution effect” when vole presence was considered. However, there was no relationship between vole density and SNV prevalence in deermice. They also found a positive relationship between vole and deermouse densities. However, these studies used subsets of data in Clay et al. (2009a) and Luis et al. (2018), which support our conclusions (see discussion in Supporting Information).

As mentioned, host regulation could affect outcomes through various components of *R*_0_. The most likely and studied mechanism is through density‐dependent transmission—when increases in host density lead to increases in intraspecific contact rates (*β*_c_[*N*]). Density‐dependent transmission should lead to a positive relationship between host density and infection prevalence. In the deermouse–SNV system, a simultaneous positive relationship between host density and infection prevalence has been found inconsistently (Clay et al. 2009a; Dizney and Ruedas 2009; Lehmer et al. 2012) leading some to conclude that diversity could not interfere with intraspecific contacts via host regulation. However, Luis et al. (2015, 2018) showed that deermouse density does correlate positively with SNV prevalence, but with a variable time lag, making it difficult to detect when host populations fluctuate. Therefore, changes in intraspecific contacts via host regulation could influence infection outcomes.

We propose that host regulation could also affect infection outcomes via transmission probability (*β*_p_) and infectious period (*σ*) (Figure 1, blue arrows). Unlike changes in contact rates through density‐dependent transmission (*β*_c_ *N*), transmission probability and infectious period have not been investigated in a “dilution effect” context, warranting additional study.

### 4.2 Mechanism 2: Encounter Interference

In addition to affecting intraspecific contacts through density‐dependent transmission (*β*_c_ *N*), diversity may also affect contact rates (*β*_c_) among hosts, independent of *N* (Figure 1, gray arrow). Encounter interference could occur with either density‐ or frequency‐dependent transmission, but due to changes in host behavior that can affect intraspecific contacts, which lead to transmission independent of changes in host density. To determine if diversity interferes with contact rates directly (*β*_c_), density‐mediated changes in contact rates must be controlled or eliminated (Figure 1, leftmost blue arrow). When diversity directly reduces or increases contacts (e.g., via host behavior), negative or positive diversity‐disease patterns can emerge. Five studies have tested for encounter interference (Table 1).

The first was from Utah, USA and found that intraspecific contact rates were lower at sites with higher diversity while accounting for host density (Clay et al. 2009b). The second was also from Utah, USA, and found that at the less diverse site deermice exhibited behaviors that could increase contacts (Dizney and Dearing 2016). The third was from Montana, USA, which reported that scar numbers of deermice were associated with densities of heterospecific competitors while accounting for host density (Eleftheriou et al. 2021). The fourth from Mexico found that diversity did not influence deermouse contacts in outdoor enclosures (Rubio et al. 2017). Lastly, Luis et al. (2018) found that for a given host density, the SNV transmission rate, *β* (product of *β*_c_ and *β*_p_), positively associated with diversity in Montana and Southwest U.S.A.

While these studies have strengths and weaknesses (see Supporting Information for discussion), they provide intriguing, yet inconclusive evidence, about whether diversity can interfere with *β*_c_ independent of *N*. We still require studies to determine whether encounter interference occurs.

### 4.3 Mechanism 3: Transmission Interference

Transmission interference occurs when diversity affects the probability of transmission given contact (*β*_p_) that depends on host susceptibility to infection and infectiousness, both of which are regulated by physiological immunity. However, studies that test transmission interference independent of *N*, must account for changes via host regulation (Figure 1, blue arrow), regardless of transmission mode. When diversity directly decreases or increases *β*_p_, negative or positive diversity‐disease patterns can emerge, respectively (Figure 1, gray arrow).

Two studies indirectly investigated transmission interference (Table 1). The first by Luis et al. (2018), could not identify whether encounter or transmission interference was responsible for a “component amplification effect”—an increase in *β* with higher diversity. The second by Eleftheriou et al. (2021), reported that densities of heterospecific competitors, but not deermouse, were associated with host stress physiology, which may impact transmission probability. Thus, there is some, yet limited evidence, to suggest whether transmission interference plays a role.

### 4.4 Mechanism 4: Infectious Period Regulation

Because reservoir hosts never recover from hantavirus infection, infected host survival (and no recovery) determines the infectious period (*σ*). Therefore, when diversity regulates infectious period by directly reducing infected host survival, it can then shorten the infectious period and lead to a negative diversity‐disease pattern. Alternatively, diversity could extend survival and lead to a positive pattern (Figure 1, gray arrow). In either case, regulation of *σ* independent of *N* could serve as a potential mechanism. If diversity also regulates *N*, it may be difficult to unravel effects of *N* via *σ* (Figure 1, blue arrow).

One study tested for infectious period regulation as a potential mechanism. It provided support for a negative relationship between diversity and deermouse density but used deermouse persistence as a measure of infectious period, which makes separating survival from dispersal challenging (Clay et al. 2009a).

## 5 Evidence From Other Rodent‐Hantavirus Systems

Additional support for host regulation as the key mechanism across rodent‐hantavirus systems comes from three larger quantitative analyses, which include systems beyond the western continental USA. The first is by Vadell et al. (2020), with disease systems from the Americas, and the second by Milholland et al. (2019), with disease systems from Asia, Europe, and the Americas, both of which centered on studies that primarily did not test for a diversity‐disease relationship but also included studies we found (Table 1). The first investigated the effect of diversity (richness and evenness) on infection prevalence and found no patterns after accounting for host density. The second examined the effect of richness and phylogenetic diversity on infection prevalence and found a negative diversity‐disease pattern, but host density was not considered. The third by Rubio et al. (2014), with systems from the Americas, found that as fragmented habitats shrunk, richness decreased and reservoir host abundance (e.g., *Peromyscus* spp.) increased, providing support for host regulation. Hence, host regulation appears to serve as the key mechanism across rodent‐hantavirus systems.

Further evidence comes from empirical studies of rodent‐hantavirus systems in Central and South America (Table 1). When diversity reduced host density, there was a negative diversity‐disease pattern (Suzán et al. 2009). When diversity was unrelated to host density, there was no pattern (Yahnke et al. 2001; Eastwood et al. 2018; Rubio et al. 2019). Although infection outcomes were not assessed, Suzán et al. (2008) from Central America also found a negative relationship between diversity and host density. Again, host regulation appears to be a key mechanism.

## 6 Conclusions

Although we acknowledge that our systematic review identified only a small number of rodent‐hantavirus systems in the Americas, we argue that when species diversity loss is driven by anthropogenic disturbance and a diversity‐disease pattern manifests, host regulation is likely to be the key causative mechanism. When diversity regulates the host population (a negative relationship), a negative diversity‐disease pattern ensues. When it does not, there is no pattern. This argument may apply in rodent‐hantavirus systems when transmission among reservoir hosts is primarily direct and density‐dependent. Not all directly transmitted disease systems meet the criteria required to demonstrate a diversity‐disease pattern (e.g., sexually transmitted diseases of humans). For those that do, rodent‐hantavirus systems are good representative models because hosts vary in their reservoir competence and the most competent hosts tend to persist as species diversity declines due to anthropogenic disturbance (Keesing and Ostfeld 2021b).

Our conclusion that host regulation is the key mechanism in directly transmitted disease systems also appears to be reflected in systems where transmission is indirect. For example, host regulation was significant in systems with environmental transmission, such as frog‐trematode (Johnson et al. 2013) and zooplankton‐fungus (Strauss et al. 2015, 2018) and vector‐borne transmission, such as birds‐West Nile virus (Swaddle and Calos 2008), rodents‐*Bartonella* bacteria (Bai et al. 2009), and Lyme disease (Levi et al. 2012).

### 6.1 Future Directions

Despite our claim that host regulation is a key mechanism, we know little about what downstream mechanisms are affected (Figure 1, blue arrows). There are also few studies that examine encounter interference, transmission interference, or infectious period regulation. Hence, we need studies that investigate these neglected mechanisms, especially when host regulation becomes less significant. For example, Luis et al. (2018) demonstrated with the deermouse‐SNV system that negative and positive diversity‐disease patterns can manifest simultaneously via different, yet competing mechanisms. Further, Kocher et al. (2023) found concurrent negative and positive diversity‐disease patterns in a vector‐borne disease system (sand flies and *Leishmania* protozoa) where higher mammal diversity decreased reservoir host density but increased sand fly density. Thus, to guide empirical investigations, we recommend that researchers consider when and what mechanisms, as described herein, may occur, and possibly interact to affect net outcomes in their own systems regardless of transmission mode.

Going forward, to understand roles of mechanisms independent of host regulation, we must design studies where we control for host density (through experiments or analyses), as we evaluate host behavior, physiology, infectious period, or their combinations. However, we recommend careful a priori consideration of what species could act on infection outcomes as well as the time of year and location. Using this approach, researchers can strategically sample the most appropriate community when detection of relevant mechanisms will be the greatest.

Methodological strategies for assessing mechanisms other than host regulation exist but can be challenging. Evaluating contact rates in wild animals using fluorescent powder marking, infrared cameras, and passive integrated transponder tags can allow for behavioral assessments, but they do come with limitations (Dearing et al. 2015). Genomic approaches where pathogens or host‐associated microbiota are tracked may also prove to be valuable in inferring contacts (Gardy and Loman 2018). Evaluating host physiology to quantify transmission probability can be even more problematic but tools from physiological ecology can help guide methodological approaches (Demas et al. 2011; Downs et al. 2014; Madliger et al. 2018). Integrating relevant physiological measures in an allostatic load index (Edes et al. 2018) or a response matrix for multivariate analyses (Telemeco and Gangloff 2020) may also be useful. Lastly, evaluating host infectious period will necessitate quantifying survival of individuals while accounting for detection probability (e.g., Eleftheriou et al. 2022). Although many technical and logistical constraints abound, these approaches will be instrumental in identifying key mechanisms.

### 6.2 Links to General Theory

Given our findings, it is helpful to ask more broadly how species diversity may affect host density. To do this, we must first consider the non‐random order species are lost as species diversity declines from anthropogenic disturbance. A negative diversity‐disease pattern generally occurs in rodent‐hantavirus systems because hosts, such as deer mice, display a specific cluster of traits: they tend to be habitat generalists, resilient to disturbance, and vulnerable to competition, predation, or both. When species diversity declines due to anthropogenic disturbance, reservoir hosts persist and are likely released from regulatory pressure, leading to population growth and higher infection prevalence through density‐dependent transmission (Mendoza et al. 2020). However, not all reservoirs are resilient, such as the pygmy rice rat (*O. longicaudatus*, ANDV reservoir), which seems to disappear from anthropogenic habitats (Rubio et al. 2019). Therefore, hantavirus reservoirs may respond differently to anthropogenic disturbance of various types, such as habitat fragmentation and agricultural expansion, which will impact the direction of a diversity‐disease pattern (Muylaert et al. 2019; García‐ Peña and Rubio 2024).

Although host reservoirs may respond differently towards anthropogenic disturbance, we may generally expect a negative diversity‐disease pattern to occur in systems where the primary host is resilient to disturbance and vulnerable to competition, predation, or both. A good example is the multi‐mammate mouse ( *Mastomys natalensis* ), the primary reservoir of Lassa virus, a mammarenavirus endemic in West Africa, because it is resilient to disturbance and vulnerable to competition, predation, or both (Jackson and Van Aarde 2004; Sluydts et al. 2009), suggesting a negative diversity‐disease pattern may occur via host regulation. However, this may not be observed when reservoirs are resilient but not vulnerable. For example, in the *Mycobacterium tuberculosis* complex (MTC) system, the most competent reservoir is the wild boar ( *Sus scrofa* ), which is resilient to disturbance but not vulnerable to competition. Barasona et al. (2019) found that ungulate diversity had no effect on boar density and consequently, there was no relationship between diversity and infection prevalence in boars. Thus, when the primary host is resilient to disturbance and vulnerable to competition, predation, or both, host regulation may lead to a negative diversity‐disease pattern. When the reservoir is resilient but not vulnerable, there may be no pattern.

In rodent‐hantavirus systems, we may observe no diversity‐disease pattern when the “diversity” measure is not regulatory—species in more diverse communities are not competitors or predators that regulate the host. For example, Orrock et al. (2011) found that predator and not competitor diversity, was negatively associated with SNV prevalence. Thus, the measure of “diversity” matters because a negative diversity‐disease pattern may not be seen if the measure is not a good proxy of species that regulate the host. For example, in the Lyme disease system, whose primary reservoir is the white‐footed mouse, a negative diversity‐disease pattern may not be observed if the “diversity” measure includes top carnivores instead of mesocarnivores, such as red foxes ( *Vulpes vulpes* ), because the latter are more likely to regulate the host (Levi et al. 2012). Furthermore, in frog‐trematode systems, richness of non‐intraguild predators reduced infections but richness of intraguild predators did not (Rohr et al. 2015).

Even primary reservoirs, such as deermice, typically regulated by “diversity” are not always across space and time. When favorable conditions (e.g., higher primary productivity) reduce interspecific competition, a diversity‐disease pattern may not manifest via host regulation and other mechanisms may rise in importance. For example, in the European bank vole‐PUUV system, although shrews are inferior competitors, they prey upon vole nestlings. Despite simultaneous rises in shrew and vole densities, a negative diversity‐disease pattern may have ensued because greater shrew predation of nestlings may have reduced intraspecific vole encounters if more time was spent at the nest (Khalil et al. 2016). Hence, when favorable environmental conditions reduce regulatory pressure, host regulation may weaken while other mechanisms may strengthen.

The concept that reservoir hosts are more resilient to disturbance has been discussed in connection with host competence. Reservoirs that are more resilient tend to be more competent for pathogens because they may allocate more resources towards reproduction than survival—that is, they follow a faster life‐history strategy (Keesing and Ostfeld 2021a). Here, we propose adding another trait dimension, vulnerability to competition, predation, or both and thus, regulatory pressure. Although competitive ability of the primary host has been identified as a key trait via empirical studies with environmentally transmitted pathogens (Hall et al. 2009; Strauss et al. 2015, 2018; Searle et al. 2016), here we extend and generalize its relevance across systems.

The question now that follows is what species are more likely to be regulated by “diversity” regardless of disease system. The literature suggests that these will typically be non‐migratory species with a wider niche breadth and smaller body size (Munday et al. 2001; Sinclair 2003). Such species tend to persist and proliferate in less diverse communities because anthropogenic disturbance favors reductions in specialist species at higher trophic levels with a larger body size (Holt et al. 1999; Elmqvist et al. 2003). Niche theory predicts that as biodiversity declines, species resilient to disturbance will expand their realized niche and grow through density compensation (Gonzalez and Loreau 2009). Thus, when reservoir hosts are mainly regulated by “diversity” (and not “bottom‐up” factors), non‐random biodiversity loss may reduce regulatory pressure and lead to a negative diversity‐disease pattern.

## 7 Final Remarks

Although we acknowledge that our conclusions stem from a relatively limited sample size of primary rodent‐hantavirus studies in the Americas, our systematic review provides novel insights by extending and refining mechanisms, complementing primary evidence with secondary studies, and providing recommendations founded in mechanistic theory. Our conclusions can be tested in rodent‐hantavirus systems outside the Americas (such as systems in Europe and Asia) and other directly transmitted disease systems. Additionally, by extending our conclusions to systems with indirect transmission, we seek to motivate a unified and integrated framework.

In conclusion, we must expand our mechanistic knowledge to effectively predict the patterns between species diversity and infection risk, particularly for directly transmitted pathogens. We recommend that key mechanisms are investigated in tandem and “diversity” measures are carefully selected. We hope that our approach and conclusions will inspire and guide empirical efforts to inform diversity‐disease theory and manage infectious diseases of animals and humans.

## Author Contributions

**Andreas Eleftheriou:** conceptualization (equal), data curation (lead), formal analysis (lead), investigation (lead), methodology (lead), project administration (lead), writing – original draft (lead), writing – review and editing (lead). **Angela D. Luis:** conceptualization (equal), investigation (supporting), methodology (supporting), project administration (supporting), supervision (lead), writing – review and editing (supporting).

## Conflicts of Interest

The authors declare no conflicts of interest.

## Supporting information


Appendix S1.

Appendix S2.

## Data Availability

This is a review paper and no primary data were collected. Raw data that were analyzed are provided in the supplemental information. Information regarding use of a software program is provided in the supplemental information.
