# Supplementary material for: Unraveling the Mechanistic Links Between Species Diversity and Infection Risk From Zoonotic Pathogens With Direct Transmission Among Reservoir Hosts: Rodent‐Orthohantavirus Systems as Models
Source: Ecol Evol. 2025 Jun 14;15(6):e71597. doi: 10.1002/ece3.71597 (PMC12166380; doi:10.1002/ece3.71597)
Supplement: Supplementary file 2 — Appendix S2. [file ECE3-15-e71597-s002.docx]

# SUPPLEMENTARY INFORMATION

*Andreas Eleftheriou, Angela Luis. Unraveling the mechanistic links between species diversity and infection risk from zoonotic pathogens with direct transmission among reservoir hosts: Rodent-orthohantavirus systems as models*

## Literature review methods

We systematically searched the literature for articles published in English with the Google Scholar search engine using the following key words: “dilution effect”, “hantavirus”, “disease”, “infection”, “rodent” and “species diversity” through April 15^th^, 2024. We also searched through reference lists of the six reviews identified through our original search and mentioned in the main text, to find studies we may have missed (*N* = 5, see Figure S1). PRISMA guidelines were followed during the literature review (see Figure S1, Page et al. 2021).

The literature search generated 379 results. Records were included for screening if they (1) were peer-reviewed empirical studies based on field studies in the Americas, and (2) focused on relationships between infection risk (antibody prevalence, nucleic acid presence or both in a primary reservoir host) and species diversity (or community composition) in rodent-hantavirus systems with or without mechanistic evidence. Given these criteria, studies that examined infection outcomes based on samples from a combination of primary and secondary reservoirs (e.g. assemblage-wide antibody prevalence) were excluded. All these criteria were initially checked by reading through the record’s title. Next, we screened all acceptable records by reading through their abstract (*N* = 52). For records deemed eligible after reading the abstract, the entire article was retrieved and evaluated (*N* = 27). Out of the five records identified through the six reviews, two were still eligible after evaluating the entire article and were included in the review. In the end, the total number of eligible records included in the review was 21.

**Identification of studies for systematic review**

Records removed *before screening*: (n = 327)

Records identified from:

Google Scholar (n = 379)

**Identification**

Records screened

(n = 52)

Records excluded

(n = 25)

Reports sought for retrieval

(n = 27)

Reports not retrieved

(n = 0)

**Screening**

Reports excluded:

(n = 11)

Reports assessed for eligibility

(n = 32)

Eligible records identified from six reviews found during initial search

(n = 5)

Studies included in review

(n = 21)

**Included**

Figure S1. Flowchart according to PRISMA guidelines detailing how the systematic review was conducted using Google Scholar engine. Flowchart was modified from the template available on <http://www.prisma-statement.org/>.

## Findings from literature review and statistical analysis

Our systematic literature review identified 21 empirical studies, including mechanistic and non-mechanistic studies. When infection outcomes or host regulation were not explicitly tested and data were accessible, we conducted our own analyses (Table S1). An additional three (not included in the 21 studies mentioned above) were quantitative analyses of multiple studies (Rubio et al. 2014, Milholland et al. 2019, Vadell et al. 2020). Four empirical studies were experimental (Suzán et al. 2009, Rubio et al. 2017, Camp et al. 2021, Spruill-Harrell et al. 2021) and some studies used subsets of the same datasets (*N* = 6, Table S2).

Most studies found no patterns (*N* = 9), and others reported negative (*N* = 5), or positive (*N* = 1) diversity-disease patterns. Each of the remaining studies (*N* = 3) reported two different outcomes depending on location and metrics (Table S2). One study reported conditional outcomes: a “component amplification” effect with overall dilution or no effect depending on geographic location. The other two studies reported dilution or no effect depending on the metric of diversity (*N* = 1) or disease (*N* = 1). There were a few studies (*N* = 5) where relationships between measures of diversity and infection or density were not statistically examined with simple linear regression. When the raw data were easily accessible in the manuscript, we statistically tested for these relationships ourselves. These studies are denoted as such in Table S2.

We conducted simple linear regression analyses in R (ver. 2022.07.2, R Core Team 2022) within RStudio (ver. 4.2.2, RStudio Team 2020). The dependent variable was either infection prevalence or host density and the independent variable was species diversity. Statistical significance was set at α = 0.05.

## Statistical results

Across all five studies, none of the linear regressions were statistically significant (Table S1). For the Clay et al. (2009b) study, neither the relationship between Simpson’s diversity index and Sin Nombre virus (SNV) prevalence (*t_8_* = -0.734, *p* = 0.484), nor deermouse density was significant (*t_8_* = -1.361, *p* = 0.210). For the Dizney and Ruedas (2004) study, neither the relationship between Simpson’s diversity index and SNV prevalence (*t_3_* = -1.386, *p* = 0.260), nor deermouse density was significant (*t_3_* = -1.501, *p* = 0.230). For the Calisher et al. (2002) study, neither the relationship between species richness and SNV prevalence (*t_1_* = -3.580, *p* = 0.173), nor deermouse density was significant (*t_1_* = 0.342, *p* = 0.790). For the Yahnke et al. (2001) study, neither the relationship between species richness and (Laguna Negra virus) LGNV prevalence (*t_2_* = -0.386, *p* = 0.737), nor small vesper mouse density was significant (*t_2_* = 0.794, *p* = 0.510). Lastly, for the Eastwood et al. (2018) study, neither the relationship between Shannon’s diversity index and (Jabora virus) JABV prevalence (*t_5_* = -0.906, *p* = 0.406), nor montane grass mouse density was significant (*t_5_* = -0.158, *p* = 0.881).

## Host regulation

Unlike Dizney and Dearing (2016), Clay et al. (2009a) found both a negative diversity-disease pattern and host regulation by using data collected over a longer time span from 16 sites. Because the study by Dizney and Dearing (2016) lacked site replication, their findings remain comparatively equivocal. Carver et al. (2011) found no relationship between concurrent SNV prevalence and vole density (i.e., no effect) at one site in Montana, U.S.A., but they did find lower SNV prevalence when voles were present. In contrast to Carver et al. (2011), Luis et al. (2018) detected no effect and no relationship between deermouse density and species diversity when they analyzed five more sites from Montana, U.S.A. However, Luis et al. (2018) compared multiple sites that had varied average values for density and diversity, whereas Carver et al. (2011) examined values within one site over time. Because of variable time lags between deermouse density and SNV prevalence (Luis et al. 2015), comparing simultaneous diversity and density may not adequately address the effects of time lags between disease and density. In summary, differences in study design and data analysis could be why these two studies do not fit the overall pattern.

Host regulation could cause dilution or amplification by affecting multiple parameters of *R_0_*. The most likely and studied mechanism is through density-dependent transmission, which if present, should lead to a positive relationship between host density and infection prevalence. In the deermouse-SNV system, researchers have not consistently found a simultaneous positive relationship between host density and infection prevalence (Clay et al. 2009a; Dizney and Ruedas 2009; Lehmer et al. 2012 but see Calisher et al. 2002), and some concluded that diversity could not interfere with intraspecific contacts via changes in host density. However, Luis et al. (2015, 2018) showed that deermouse density does correlate positively with SNV prevalence, but with a variable time lag, making it difficult to detect when host populations fluctuate. Given strong support for density-dependent transmission, diversity-mediated changes in intraspecific contacts via host regulation could indeed have a significant influence on infection prevalence.

## Encounter interference

Two studies (Clay et al. 2009b, Eleftheriou et al. 2021) were performed at sites naturally varying in deermouse density. The first study is from Utah, U.S.A., which found lower intraspecific contact rates at more diverse sites while accounting for deermouse density (Clay et al. 2009b). The authors used foraging arenas and infrared cameras to document frequency and duration of contacts. Their findings suggested that a higher diversity (Simpson’s index) decreases frequency of contacts but not their duration. Although intriguing, two limitations were the inclusion of an influential outlier, and the foraging arenas may have artificially modified behavior. The second study from Montana, U.S.A., found that the number of scars (proxy for contact rates occurring from intraspecific fighting) was differentially associated with densities of shrews (*Sorex* spp.) and voles (*Microtus* spp.), but not deermice (Eleftheriou et al. 2021). Despite interesting conclusions, number of scars is unlikely to be a very precise measure for contact rates. In summary, it remains challenging to make conclusions from these few studies about the role of encounter interference.

Two other studies (Dizney and Dearing 2016; Rubio et al. 2017) controlled for host density differently. The first was done in Utah, U.S.A., where the two sites naturally varied in rodent diversity (Shannon’s index), but not current deermouse density (Dizney and Dearing 2016). Deermice at the less diverse site exhibited behaviors that could increase contacts (e.g., time spent foraging). However, there was no site replication and they used foraging arenas, making results equivocal. The second study by Rubio et al. (2017) used experimental enclosures in Mexico and found that rodent diversity (richness and Shannon’s index) did not influence deermouse intraspecific contacts. Although they did not test deermice for SNV and used foraging arenas, they did measure *β_c_* by holding *N* constant.

The last study by Luis et al. (2018), which used field sites that naturally varied in deermouse density, found that for a given deermouse density, the transmission rate of SNV, *β* (the product of *β_c_* and *β_p_*), increased with higher small mammal diversity in both Montana and Southwest U.S.A. However, they could not disentangle whether the causative mechanism was encounter or transmission interference.

Table S1. Statistical summary from simple linear regressions using data from original studies.

| Study | Response | Predictor | *β* estimate | *t* value | *p* value |
| --- | --- | --- | --- | --- | --- |
| Clay et al. 2009b | Infection prevalence  Host density | Diversity Index | -0.184  -12.949 | -0.734  1.361 | 0.484  0.210 |
| Dizney & Ruedas 2004 | Infection prevalence  Host density | Diversity Index | -0.245  -964.400 | -1.386  -1.501 | 0.260  0.230 |
| Calisher et al. 2002 | Infection prevalence  Host density | Diversity Index | -0.006  0.161 | -3.580  0.342 | 0.173  0.790 |
| Yahnke et al. 2001 | Infection prevalence  Host density | Diversity Index | -0.015  13.420 | -0.386  0.794 | 0.737  0.510 |
| Eastwood et al. 2018 | Infection prevalence  Host density | Diversity Index | -0.132  -13.000 | -0.906  -0.158 | 0.406  0.881 |

Table S2. Diversity-disease studies (mechanistic and non-mechanistic) with rodent-hantavirus systems in the Americas. We list data on the disease system, diversity-disease outcome, support (with directionality) for relationships between diversity and host density (*N*) (mechanism = host regulation), diversity and intraspecific contact rates ($\beta_{c}$) (mechanism = encounter interference), diversity and transmission probability ($\beta_{p}$) (mechanism = transmission interference), and diversity and host infectious period (*σ*) (mechanism = infectious period regulation), metrics of diversity and disease, and study reference.

| Rodent-virus system | Disease outcome | Host density (*N*) | Contact rates ($\beta_{c}$) | Transmission  probability ($\beta_{p}$) | Host infectious period ($\sigma$) | Diversity metric | Disease metric | Study reference |  |
| --- | --- | --- | --- | --- | --- | --- | --- | --- | --- |
| *P. maniculatus*–SNV | D^a^ | - | . | . | -? | RDY | AP | Clay et al. 2009a |  |
|  | N^a^ | N | -? | . | . | RDY | AP | Clay et al. 2009b^†^ |  |
|  | N | . | . | . | . | SDY | AP | Orrock et al. 2011 |  |
|  | D | . | . | . | . | PDY | AP | Orrock et al. 2011 |  |
|  | N | N | . | . | . | SDY | AP | Dizney & Ruedas 2009^†^ |  |
|  | D | - | . | . | . | SDY | AP/NI | Lehmer et al. 2012 |  |
|  | D | N | - | . | . | RDY | AP | Dizney & Dearing 2016 |  |
|  | . | N | N | . | . | RDY | . | Rubio et al. 2017 |  |
|  | D^b^ | - | . | . | . | SDY | AP/NI | Luis et al. 2018 |  |
|  | N^c^ | N | . | . | . | SDY | AP/NI | Luis et al. 2018 |  |
|  | CA^b^  CA^c^ | *NA*  *NA* | +?  +? | +?  +? | . . | SDY  SDY | AP  AP | Luis et al. 2018  Luis et al. 2018 |  |
|  | N^b^ | N | . | . | . | RDY | AP | Calisher et al. 2002^†^ |  |
|  | N^c^ | + | . | . | . | NHD | AP | Carver et al. 2011 |  |
|  | D^b^ | . | . | . | . | SDY | AP | Mills 2006 |  |
|  | . | . | +*/*NM | + | . | NHD | . | Eleftheriou et al. 2021 |  |
| *O. longicaudatus* – ANDV | N  D | .  . | .  . | .  . | .  . | RDY  RDY | AP  NI | Piudo et al. 2011  Piudo et al. 2011 | |
|  | N | N | . | . | . | SDY | AP/NI | Rubio et al. 2019 | |
| *C. laucha* – LGNV | N | N | . | . | . | SDY | AP | Yahnke et al. 2001^†^ | |
| *A. montensis* – JABV | N | N | . | . | . | RDY | AAP | Eastwood et al. 2018^†^ | |
| *O. flavescens - various* | A | . | . | . | . | RDY | AP | Maroli et al. 2023 | |
| Various | D | - | . | . | . | RDY | AP | Suzán et al. 2009 | |
|  | . | - | . | . | . | SDY | . | Suzán et al. 2008 | |
|  | N  N | .  . | .  . | .  . | .  . | RDY  RDY | AP  AP | Camp et al. 2021  Spruill-Harrell et al. 2021 | |

RODENT-VIRUS SYSTEM: SNV = Sin Nombre virus, BAYV = Bayou virus, ANDV = Andes virus, LGNV = Laguna Negra virus, JABV = Jabora virus. DISEASE OUTCOME: D = dilution, A = amplification, CA = component amplification (i.e. not the main effect). DIVERSITY METRIC: RDY= rodent diversity, SDY = small mammal diversity, PDY = predator diversity, NHD = nonhost density. DISEASE METRIC: AP = antibody prevalence, NI = number of infected hosts, AAP = antibody and/or antigen prevalence. ALL: ‘.’ = not explicitly examined, ‘+’ = positive relationship, ‘-’ = negative relationship, N = no relationship, NM = non-monotonic, NA = not applicable. Studies that share letter superscripts (a-c) used subsets of the same datasets. Question marks denote published relationships that the authors find unclear.

^†^ Includes results from statistical analyses that were not performed by the original study authors.

## References

Calisher, C.H., Root, J.J., Mills, J.N. and Beaty, B.J. (2002) Assessment of ecologic and biologic factors leading to hantavirus pulmonary syndrome, Colorado, USA. *Croatian medical journal,***43**, 330-337.

Camp JV, Spruill-Harrell B, Owen RD, Solà-Riera C, Williams EP, Eastwood G, Sawyer AM, Jonsson CB. (2021) Mixed effects of habitat degradation and resources on hantaviruses in sympatric wild rodent reservoirs within a neotropical forest. *Viruses*. **13**, 85.

Carver, S., Kuenzi, A., Bagamian, K.H., Mills, J.N., Rollin, P.E., Zanto, S.N. and Douglass, R. (2011) A temporal dilution effect: Hantavirus infection in deer mice and the intermittent presence of voles in Montana. *Oecologia,***166**, 713-721.

Clay, C.A., Lehmer, E.M., Jeor, S.S. and Dearing, M.D. (2009a) Sin Nombre virus and rodent species diversity: A test of the dilution and amplification hypotheses. *PloS one,***4**, e6467.

Clay, C.A., Lehmer, E.M., Jeor, S.S. and Dearing, M.D. (2009b) Testing mechanisms of the dilution effect: Deer mice encounter rates, Sin Nombre virus prevalence and species diversity. *EcoHealth,***6**, 250-259.

Dizney, L. and Dearing, M.D. (2016) Behavioural differences: A link between biodiversity and pathogen transmission. *Animal Behaviour,***111**, 341-347.

Dizney, L.J. and Ruedas, L.A. (2009) Increased host species diversity and decreased prevalence of Sin Nombre virus. *Emerging infectious diseases,***15**, 1012-1018.

Eastwood, G., Camp, J.V., Chu, Y.K., Sawyer, A.M., Owen, R.D., Cao, X., Taylor, M.K., Valdivieso-Torres, L., Sage, R.D. and Yu, A. (2018) Habitat, species richness and hantaviruses of sigmodontine rodents within the interior Atlantic forest, Paraguay. *PloS one,***13**, e0201307.

Eleftheriou, A., Kuenzi, A.J., and Luis, A.D. (2021) Heterospecific competitors and seasonality affect host physiology and behavior; key determinants of disease transmission. *Ecosphere*, **12**, e03494.

Lehmer, E.M., Korb, J., Bombaci, S., McLean, N., Ghachu, J., Hart, L., Kelly, A., Jara-Molinar, E., O’Brien, C. and Wright, K. (2012) The interplay of plant and animal disease in a changing landscape: The role of sudden aspen decline in moderating Sin Nombre virus prevalence in natural deer mouse populations. *EcoHealth,***9**, 205-216.

Luis, A.D., Douglass, R.J., Mills, J.N. and Bjørnstad, O.N. (2015) Environmental fluctuations lead to predictability in Sin Nombre hantavirus outbreaks. *Ecology,***96**, 1691-1701.

Luis, A.D., Kuenzi, A.J. and Mills, J.N. (2018) Species diversity concurrently dilutes and amplifies transmission in a zoonotic host-pathogen system through competing mechanisms. *Proceedings of the National Academy of Sciences of the United States of America,***115**, 7979-7984.

Maroli, M., Bellomo C.M., Coelho R.M., Martinez V.P., Piña C.I., Gómez Villafañe I.E. 2023. Orthohantavirus infection in two rodent species that inhabit wetlands in Argentina. *EcoHealth*, **20,** 402-415.

Milholland, M.T., Castro-Arellano, I., Garcia-Peña, G.E. and Mills, J.N. (2019) The ecology and phylogeny of hosts drive the enzootic infection cycles of hantaviruses. *Viruses,***11**, 671.

Page, M. J., McKenzie, J. E., Bossuyt, P. M., Boutron, I., Hoffmann, T. C., Mulrow, C. D., ... & Moher, D. (2021). The PRISMA 2020 statement: an updated guideline for reporting systematic reviews. *International journal of surgery*, **88**, 105906.

R Core Team, 2022. R: A language and environment for statistical computing. R Foundation for Statistical Computing, Vienna, Austria. URL <https://www.R-project.org/>.

RStudio Team, 2020. RStudio: Integrated Development Environment for R. RStudio, PBC, Boston, MA. URL <http://www.rstudio.com/>.

Rubio, A.V., Castro-Arellano, I., Mills, J.N., List, R., Ávila-Flores, R. and Suzán, G. (2017) Is species richness driving intra-and interspecific interactions and temporal activity overlap of a hantavirus host? an experimental test. *PloS one,***12**, e0188060.

Rubio, A. V., Ávila-Flores, R. and Suzán, G. (2014) Responses of small mammals to habitat fragmentation: epidemiological considerations for rodent-borne hantaviruses in the Americas. *EcoHealth*. **11**, 526-533.

Spruill-Harrell B, Pérez-Umphrey A, Valdivieso-Torres L, Cao X, Owen RD, Jonsson CB. (2021). Impact of Predator Exclusion and Habitat on Seroprevalence of New World Orthohantavirus Harbored by Two Sympatric Rodents within the Interior Atlantic Forest. *Viruses.* **13**, 1963.

Suzán G, Marcé E, Giermakowski JT, Mills JN, Ceballos G, Ostfeld RS, Armién B, Pascale JM, Yates TL. (2009). Experimental evidence for reduced rodent diversity causing increased hantavirus prevalence. *PloS one.* **4**,e5461.

Vadell, M.V., Villafañe, I.E.G. and Carbajo, A.E. (2019) Hantavirus infection and biodiversity in the Americas. *Oecologia,* **192**, 169-177.

Yahnke, C.J., Meserve, P.L., Ksiazek, T.G. & Mills, J.N. (2001) Patterns of infection with Laguna Negra virus in wild populations of *Calomys laucha* in the central Paraguayan Chaco. *The American Journal of Tropical Medicine and Hygiene,***65**, 768-776.
